# Cotton and Surgical Face Masks in Community Settings: Bacterial Contamination and Face Mask Hygiene

**DOI:** 10.3389/fmed.2021.732047

**Published:** 2021-09-03

**Authors:** Lize Delanghe, Eline Cauwenberghs, Irina Spacova, Ilke De Boeck, Wannes Van Beeck, Koen Pepermans, Ingmar Claes, Dieter Vandenheuvel, Veronique Verhoeven, Sarah Lebeer

**Affiliations:** ^1^Department of Bioscience Engineering, University of Antwerp, Antwerp, Belgium; ^2^Faculty of Social Sciences, University of Antwerp, Antwerp, Belgium; ^3^Department of Primary and Interdisciplinary Care (FAMPOP), University of Antwerp, Antwerp, Belgium

**Keywords:** SARS-CoV-2, COVID-19, face masks, bacterial load, 16S rRNA gene amplicon sequencing, nasal and skin microbiome

## Abstract

During the current COVID-19 pandemic, the use of face masks has become increasingly recommended and even mandatory in community settings. To evaluate the risk of bacterial cross-contamination, this study analyzed the bacterial bioburden of disposable surgical masks and homemade cotton masks, and surveyed the habits and face mask preferences of the Flemish population. Using culture approaches and 16S rRNA gene amplicon sequencing, we analyzed the microbial community on surgical and/or cotton face masks of 13 healthy volunteers after 4 h of wearing. Cotton and surgical masks contained on average 1.46 × 10^5^ CFU/mask and 1.32 × 10^4^ CFU/mask, respectively. *Bacillus, Staphylococcus*, and *Acinetobacter* spp. were mostly cultured from the masks and 43% of these isolates were resistant to ampicillin or erythromycin. Microbial profiling demonstrated a consistent difference between mask types. Cotton masks mainly contained *Roseomonas, Paracoccus*, and *Enhydrobacter* taxa and surgical masks *Streptococcus* and *Staphylococcus*. After 4 h of mask wearing, the microbiome of the anterior nares and the cheek showed a trend toward an altered beta-diversity. According to dedicated questions in the large-scale Corona survey of the University of Antwerp with almost 25,000 participants, only 21% of responders reported to clean their cotton face mask daily. Laboratory results indicated that the best mask cleaning methods were boiling at 100°C, washing at 60°C with detergent or ironing with a steam iron. Taken together, this study suggests that a considerable number of bacteria, including pathobionts and antibiotic resistant bacteria, accumulate on surgical and even more on cotton face masks after use. Based on our results, face masks should be properly disposed of or sterilized after intensive use. Clear guidelines for the general population are crucial to reduce the bacteria-related biosafety risk of face masks, and measures such as physical distancing and increased ventilation should not be neglected when promoting face mask use.

## Introduction

During the current coronavirus disease 2019 (COVID-19) pandemic, caused by the severe acute respiratory syndrome coronavirus 2 (SARS-CoV-2), the use of protective face masks has become increasingly recommended or even mandatory in community settings outside hospitals and care facilities (1, 2). Surgical or cotton masks are most often used to prevent respiratory droplet transmission and reduce transmission from people infected with respiratory viruses to non-infected people (3, 4). Due to some shortage in supply and concern about excessive waste of disposable masks, policy makers promote homemade non-medical (i.e., non-surgical) masks as personal protective equipment (PPE). As opposed to medical masks (e.g., surgical, medical procedure face masks, and respirators) that represent standardized personal equipment (PPE), non-medical masks are considered as not standardized and not intended for use in healthcare professionals (5). Although there are concerns that their filter efficacy is less able to block the transmission of viruses compared to surgical masks, this could be compensated by better adjustment to the face and less leakage (6). The efficacy of face masks against different airborne transmissions is best documented in controlled settings, such as use in hospitals by trained staff (7–9). Additionally, recent research has shown that they can also reduce COVID-19 transmission in other high-risk situations, such as hospitals (2, 10, 11). One meta-analysis and systematic review has concluded that the use of masks by healthcare workers and non-healthcare workers reduces the risk of respiratory virus transmission (including SARS, influenza virus, H1N1, and SARS-CoV-2) with 80 and 47%, respectively (2). However, this significant protective effect of face masks in community and health care settings was not found by other studies (1, 7, 12–17). Additionally, a recent randomized controlled trial study observed that wearing a face mask did not significantly reduce the SARS-CoV-2 infection rate in a community with modest infection rates (18). Since masks were not recommended public health measures most of the population did not wear face masks during this clinical trial. These results were also supported by another recent meta-analysis (19). In general, masks seem less effective in protecting the wearer from being infected (20), but they could reduce the risk of virus transmission when worn consistently (10, 21, 22).

The general assumption is that both medical and non-medical mask use is safe (23, 24), although this has not yet been monitored or studied in detail. Studies on mask efficacy [e.g., (25–27)] generally do not account for the fact that the microorganisms in human saliva and exhaled breath could form a biosafety concern, especially when masks are worn for too long, not properly stored, or re-used without proper disinfection. In fact, the human saliva contains 100 million bacterial cells per milliliter and harbors a range of pathobionts, including *Staphylococcus aureus, Pseudomonas aeruginosa, Candida albicans, Klebsiella pneumoniae, Neisseria, Prevotella*, and *Veillonella spp*. (28–31). Furthermore, cotton serves as substrate for microbial growth (32) and is able to retain moisture, making cotton masks more favorable for high microbial contamination than surgical masks. In addition, the reuse of cotton masks, moisture retention and poor filtration may result in increased risks of transmission of respiratory viruses compared to surgical masks (9).

Policy makers are beginning to recognize the biosafety hazards of wearing non-professional face masks. The Belgian government recommends that after 8 h of regular use or 4 h of intensive use (e.g., intensive speaking during teaching), the face mask should be replaced or cleaned (33). Used fabric face masks should be kept in a closed cloth bag and washed together. It is recommended to wash (60°C with detergent), boil (100°C), or iron the masks to disinfect them after use. After disinfection, the mask should be completely dried before wearing it again (33). Nevertheless, the general population is not yet properly educated to handle face masks. An observational checklist with 1,500 participants recruited in Hong Kong showed that almost none of them were able to perform all the required steps in using a face mask correctly, as 91.5% did not perform hand hygiene before putting the mask on and 97.3% when taking it off (34). Improper use of face masks can lead to a higher risk of infection with and spreading of viral and bacterial pathogens. Self-inoculation of mucous membranes of nose, eyes, and mouth is an important transmission route of viruses (35), as people touch their face ~23 times per hour of which 44% involves contact with a mucous membrane (36). Moreover, people may pay less attention to other important measures such as social distancing and hand hygiene (24, 37). Lastly, face mask use can be associated with discomfort, skin acne, headaches, respiratory distress, difficulties in communication (especially for deaf or hard of hearing persons), as well as less non-verbal communication (24, 38, 39).

In this study we compared the bacterial load and microbiome composition on certified disposable surgical masks and self-made cotton masks, to evaluate some risks for bacterial cross-contamination. We also performed microbiome profiling of the cheek and anterior nares before and after wearing the face mask in order to detect shifts in the microbiome caused by face mask wearing. Particular attention was given to antibiotic-resistant bacteria, putative food-related pathogens, and skin and respiratory pathobionts. In parallel, as part of a large-scale survey conducted by the University of Antwerp, we assessed the hygiene habits, preferences, opinions, and influences on social behavior related to face masks in the Belgian population.

## Materials and Methods

### Microbiological Mask Study Design

The protocol of this study was in accordance with the Declaration of Helsinki. The study was approved by the ethical committee of the University Hospital of Antwerp (Belgium). The study was given the approval number B3002021000072 (Belgian registration) and was registered online at clinicaltrials.gov with unique identifier NCT04894422.

Healthy volunteers (four males and nine females) aged 24-33 who wore surgical (certified as medical device) and/or a self-made cotton masks (stitching pattern used is presented in Supplementary Figure 1) in an indoor setting were asked to return their mask after 4 h wearing. All healthy volunteers were non-smokers, non-healthcare workers and did not take any antibiotics, nor had a hospital stay in the previous month before the start of the study. All cotton masks were made according to the instructions recommended by the Belgian government (pattern with specific measurements can be found in Supplementary Figure 1), initially cleaned by washing at 60°C with detergent and stored in a closed ziplock bag until use. In total, 21 masks, worn for 4 h, were collected. The face masks were then cut in half using sterile scissors under sterile conditions. One half of the mask was cut in smaller pieces and put in a 50 ml conical tube. Fifteen ml of sterile phosphate-buffered saline (PBS) was added and the tube was vortexed for 30 s. The suspension was plated out in serial dilutions in PBS for bacterial colony forming unit (CFU) counts and used for DNA extraction (more details under “Microbiology methods”). The other half of the mask was cleaned in a home setting by soaking in boiling water for a few minutes (100°C), washing in a washing machine at 60°C with detergent, ironing with a steam iron for ~2 min, leaving overnight in the freezer at (avg. −18°C), or leaving at room temperature for 72 h. After treatment, the cleaned half of the mask was processed and plated out as described for the first half. To check initial bacterial load on the mask, clean and never-used cotton (5) and surgical masks (7) were plated out for bacterial loads as described previously.

Additionally, we collected a non-invasive nasal swab (anterior nares) [Nasal swab Copan (catalog number 503CS01)] and a non-invasive skin swab (cheek skin) (eNAT swab) of 10 participants before and after wearing the face mask after approval of the ethical committee (B3002021000072). All participants gave their consent before swabs were collected. Skin swabs were stored at 4°C (max 24 h) prior to DNA extraction and 500 μl of the eNAT buffer was used for the DNA extraction. Nasal swabs were immediately suspended in 750 μl MoBio bead solution (PowerFecal DNA Isolation Kit; MO BIO Laboratories Inc., Carlsbad, CA, United States) and kept at 4°C (max 24 h) until further processing.

### Bacterial Isolation and Culturing

Brain Heart Infusion (BHI) agar (LAB M, Lancashire, UK) and Lysogeny Broth (LB) agar (Carlroth, Karlsruhe, Germany) plates (composition given in Supplementary Table 2), containing 1.5% (v/w) agar, were used to determine the bacterial load on the face masks before and after wearing and cleaning. The bacterial load was determined in colony forming units per ml (CFU/ml) of resuspension medium and recalculated to CFU/mask. The face mask suspension was diluted 10 times and 100 μl of undiluted and diluted suspensions were plated out on both growth media. Plates were incubated overnight at 37°C in aerobic conditions. CFUs were counted and a total of 47 colonies were isolated, subjected to colony PCR and identified using Sanger sequencing as described below. For isolation, colonies were transferred to BHI or LB liquid medium, grown statically at 37°C overnight and used for making glycerol stocks in 25% v/v glycerol stored at −80°C. To determine antibiotic resistance, the isolates were plated out on LB or BHI agar containing ampicillin and erythromycin, each at 100 μg/mL; final concentration.

### Taxonomic Identification of Isolates by Sanger Sequencing of the 16S rRNA Gene

Grown isolates were further identified with PCR and Sanger sequencing of the 16S rRNA gene. The target region was amplified by using 27 forward primer (27f 5′-AGAGTTTGATCCTGGCTCAG-3′) and 1492 reverse primer (1492r 5′-CTACGGCTACCTTGTTACGA-3′) (40, 41). Sanger sequencing was performed at the “VIB genetic service facility” (University of Antwerp) on a capillary sequencer (Applied Biosystems 3730XL DNA Analyzer) and ABI PRISM® BigDye^TM^ Terminator cycle sequencing kit. The Sanger sequencing data was analyzed with Geneious prime (version 2020.0.5), ends of sequenced reads were trimmed with an error probability limit of 0.05, the sequences were assembled and the quality of the assemblies was assessed. Finally, all the assemblies were blasted in the NCBI database.

### Bacterial DNA Extraction From Swabs

The PowerFecal DNA Isolation Kit was used to extract microbial DNA from anterior nares and cheek swabs. DNA extraction was performed according to the instructions of the manufacturer. Negative extraction controls were extracted at regular time points throughout the study. DNA concentrations were measured using the Qubit 3.0 Fluorometer (Life Technologies, Ledeberg, Belgium).

### Illumina MiSeq 16S rRNA Gene Amplicon Sequencing

Illumina MiSeq 16S rRNA gene amplicon sequencing was performed on the extracted DNA from the nasal and skin swabs as well as on the PBS suspension of the face masks to determine the taxonomic composition of the bacterial communities. An in-house optimized protocol for low-biomass samples was followed, as described (42). Briefly, 5 μl of bacterial DNA sample was used to amplify the V4 region of the 16S rRNA gene. All DNA samples and negative controls of both PCR (PCR grade water) and the DNA extraction kit were included. Standard barcoded forward (515F) and reverse primer (806R) were used. These primers were altered for dual index paired-end sequencing, as described in (43). The resulting PCR products were checked on a 1.2% agarose gel. The PCR products were purified using the Agencourt AMPure XP Magnetic BeadCapture Kit (Beckman Coulter, Suarlee, Belgium) and the DNA concentration of all samples was measured using the Qubit 3.0 Fluorometer. Next, a library was prepared by pooling all PCR samples in equimolar concentrations, loaded onto a 0.8% agarose gel and purified using the NucleoSpin Gel and PCR clean-up (Macherey-Nagel). The final DNA concentration of the library was measured with the Qubit 3.0 Fluorometer. Afterwards the library was denatured with 0.2N NaOH (Illumina), diluted to 7 pM and spiked with 10% PhiX control DNA (Illumina). MiSeq Desktop sequencer (M00984, Illumina) was used for sequencing.

Processing and quality control of the reads were performed using the R package DADA2, version 1.6.0. Further processing of the ASV table, AVS annotation (e.g., classification), sample annotation (metadata) and data visualization and statistical analysis was performed in R version 4.0.3 (44), using the in-house package tidyamplicons, version 0.2.1 [publicly available at github.com/SWittouck/tidyamplicons), as described in (42)]. Alpha- and beta-diversity analysis were also performed as described before in (42).

### Large-Scale Survey on Face Mask Use and Attitudes

On August 19th, 2020, 24.948 people filled in the University of Antwerp Great Corona survey through the online survey system Qualtrics. Questions regarding the hygiene habits, preferences, opinions, and influences on social behavior related to face masks in the Belgian population were included. This citizen science project was organized by the Centre for Health Economics Research and Modelling Infectious Diseases (University of Antwerp), Data Science Institute (University of Hasselt), the SIMID collaboration, the Centre for the Evaluation of Vaccination (University of Antwerp) and Koen Pepermans (Faculty of Social Sciences, University of Antwerp). The Great Corona survey is supported by the Research Foundation Flanders (Grant G0G1920N, 2020).

### Statistical Analysis

Statistical analysis was performed in R for 16S amplicon sequencing data and GraphPad Prism version 8.4.3 for other data. The following statistical tests were used: Wilcoxon test and multiple *t*-tests, Kruskal-Wallis test with multiple comparisons, two-way ANOVA with Sidak's multiple comparisons test.

### Data Availability

Sequencing data are available at the European Nucleotide Archive with the accession number PRJEB45406.

## Results

### Bacterial Composition of Surgical and Cotton Face Masks After Use

**Figure 1:**
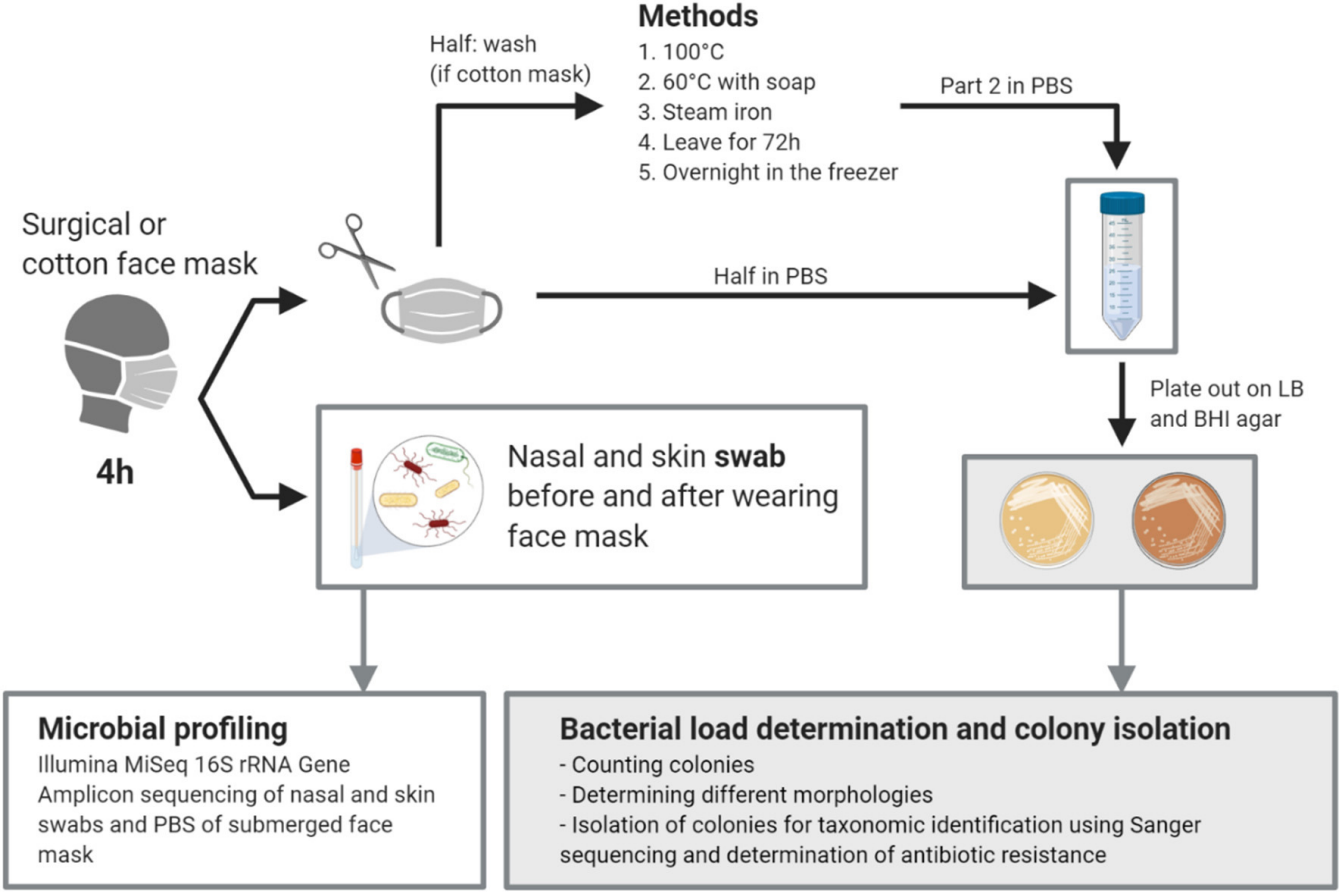
Graphical abstract of the study. Created with BioRender.com.

**Figure 2:**
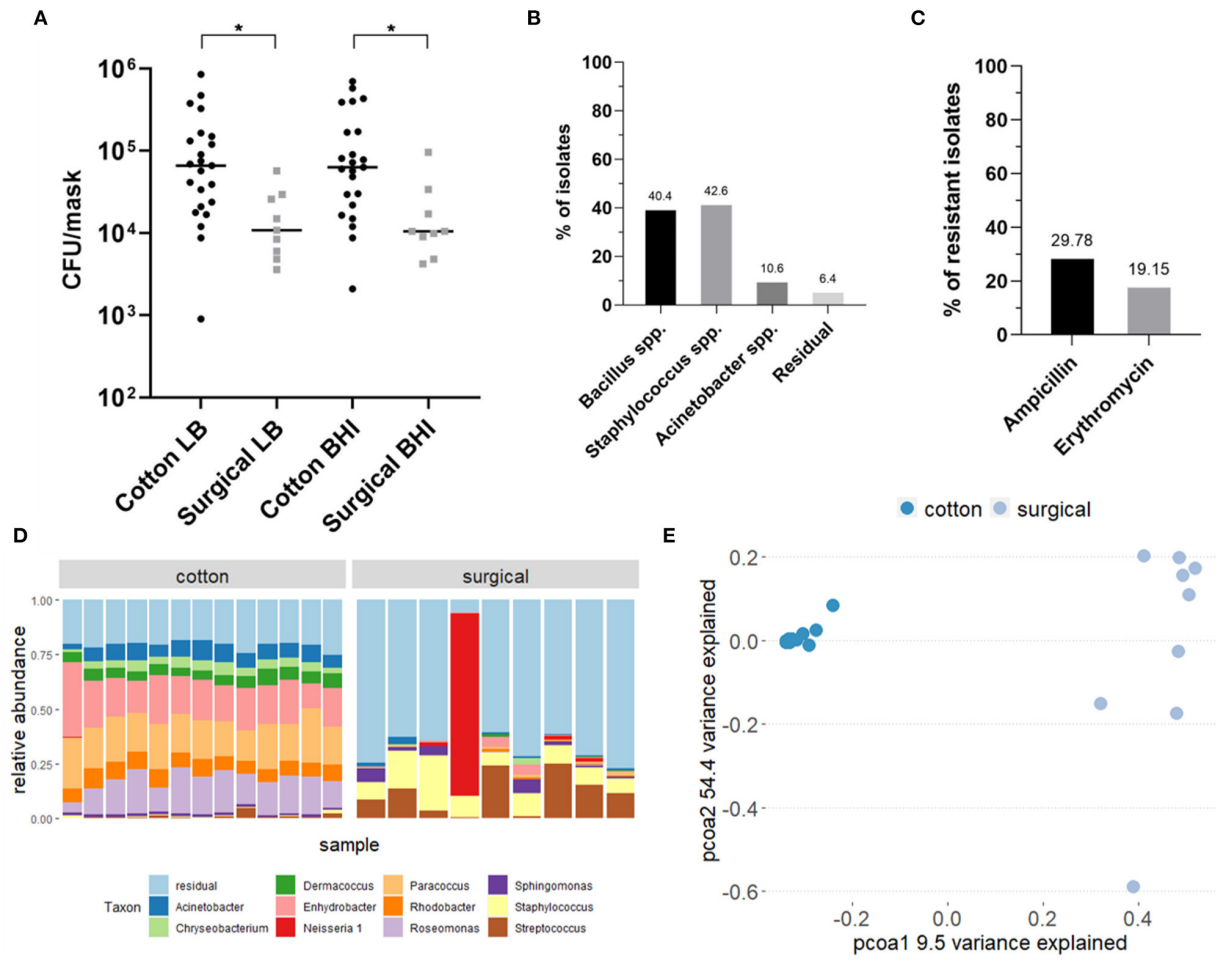
Analysis of the bacterial composition of cotton and surgical face masks after 4 h of wearing. **(A)** Mask bacterial load quantified *via* culturing on LB and BHI agar plates after 4 h of wearing. Data shown the mean CFU/mask (calculated to represent the whole face mask) for both growth media and mask types. Statistics were performed using ANOVA with Kruskal-Wallis multiple comparisons test *p < 0.05. **(B)** Identification of selected bacterial isolates cultured from both types of face masks through Sanger sequencing of the V4 region of the 16S rRNA gene. **(C)** Percentages of isolates cultured from both types of face masks that showed resistance to the antibiotics ampicillin and erythromycin. **(D)** Taxonomic bacterial community composition on the face masks at genus-level where each bar represents a participant, analyzed *via* 16S rRNA amplicon sequencing. **(E)** Principal Coordinates Analysis (PCoA) plot distributing the samples according to beta-diversity (Bray-Curtis distance). Samples are colored by type of mask. LB, Luria-Bertani broth; BHI, Brain Heart Infusion; AB, antibiotic; C, cotton face mask; S, surgical face mask.

An overview of the study design can be found in the graphical abstract in Figure 1. In total, the bacterial load and composition of 23 cotton and 9 surgical masks, worn for 4 h by the participants, was determined. The initial bacterial load of clean and never used cotton face masks was 1.44 × 10^3^ CFU/mask (mean, SD = 1.09 × 10^3^) on Lysogeny Broth (LB) agar and 1.50 × 10^3^ CFU/mask (mean, SD = 1.51 × 10^3^) on Brain Heart Infusion (BHI) agar (average of 1.47 × 10^3^ CFU/mask). For the surgical masks, the initial load was 1.29 × 10^2^ CFU/mask (mean, SD = 1.60 × 10^2^) on LB and 4.29 × 10^1^ CFU/mask (mean, SD = 1.31 × 10^2^) on BHI (average of 8.60 × 10^1^ CFU/mask). After 4 h of wearing, the cotton masks contained a mean of 1.38 × 10^5^ CFU/mask (SD = 1.95 × 10^5^) counted on LB and 1.53 × 10^5^ CFU/mask (SD = 1.96 × 10^5^) on BHI growth medium (average of 1.46 × 10^5^ CFU/mask). The surgical masks contained a mean of 1.79 × 10^4^ CFU/mask (SD = 1.63 × 10^4^) on LB and 2.18 × 10^4^ CFU/mask (SD = 2.76 × 10^4^) on BHI (average of 1.32 × 10^4^ CFU/mask). So after wearing, the cotton masks contained significantly more CFU/mask for both growth media (*p* = 0.021, LB; *p* = 0.014, BHI) (Figure 2A). However, a higher increase in bacterial load compared to the initial bacterial load was seen for surgical masks. Overall, high interindividual variation in the bacterial load on the face masks after wearing was seen, represented in the high standard deviations.

Based on colony morphology, we selected 47 individual colonies from across all mask types for identification using 16S rRNA gene-based Sanger sequencing. Most colonies were identified as *Bacillus* or *Staphylococcus* species, comprising, respectively, 40.4 and 42.6% of all selected colonies (Figure 2B). Among the *Bacillus* species, *Bacillus thuringiensis* and *Bacillus cereus* were most represented (Supplementary Table 1). Among the *Staphylococcus* species, we found mostly *Staphylococcus epidermidis*, as well as *Staphylococcus aureus, Staphylococcus warneri*, and *Staphylococcus caprae*, which are known species of a healthy human skin and nasal microbiome (42, 45–47). Furthermore, 10.6% of all colonies was identified as *Acinetobacter* spp., which are also considered a part of the normal human skin and respiratory microbiome (48, 49). Along with the three most abundant isolated species, residual isolates included *Pantoea, Lysinibacillus*, and *Solibacillus* species.

Antibiotic-resistant bacteria are a worldwide problem as infections caused by these micro-organisms are more difficult to treat and can lead to higher medical costs, prolonged hospital stays and increased mortality. Therefore, we tested the resistance of all bacterial isolates to two commonly used antibiotics for respiratory infections: erythromycin and ampicillin, a macrolide and beta-lactam antibiotic, respectively. Both antibiotics are active against important respiratory pathogens and resistance to both is increasing (50–55). Of the 47 isolated colonies, 43% showed full antimicrobial resistance to at least one of the tested antibiotics: 30% of the isolates were resistant against ampicillin and 19% against erythromycin (Figure 2C). Six percent of all colonies showed resistance against both antibiotics.

In addition to cultivation-based methods, we analyzed the microbiome composition of masks using 16S rRNA gene amplicon sequencing (Figure 2D). Not-worn face masks were not used in this step as the amount of bacterial cells was too low to be detected using this method. The bacterial community residing in the cotton face masks after 4 h of wearing were mainly represented by *Roseomonas, Paracoccus*, and *Enhydrobacter*, with mean relative abundances of 15.23, 19.00, and 19.28%, respectively. In contrast, the microbial communities found on the surgical masks consisted mostly of *Streptococcus* (mean relative abundance 11.31%) and *Staphylococcus* (mean relative abundance 11.03%). These genera are also present on the cotton masks, but in lower relative abundances, 4% for *Staphylococcus* and 3% for *Streptococcus*.

In order to explore the differences in beta-diversity (measured in terms of Bray-Curtis distance) between the cotton and surgical face masks, Principal Coordinates Analysis (PCoA) was used. Based on the type of mask, two clear clusters could be observed presenting either the cotton or surgical masks (Figure 2E). A significant impact of the type of face mask on the alpha diversity was observed, measured with inverse Simpson index (*p* = 0.0033, Supplementary Figure 3).

### Cleaning Methods for Cotton Face Masks

**Figure 3:**
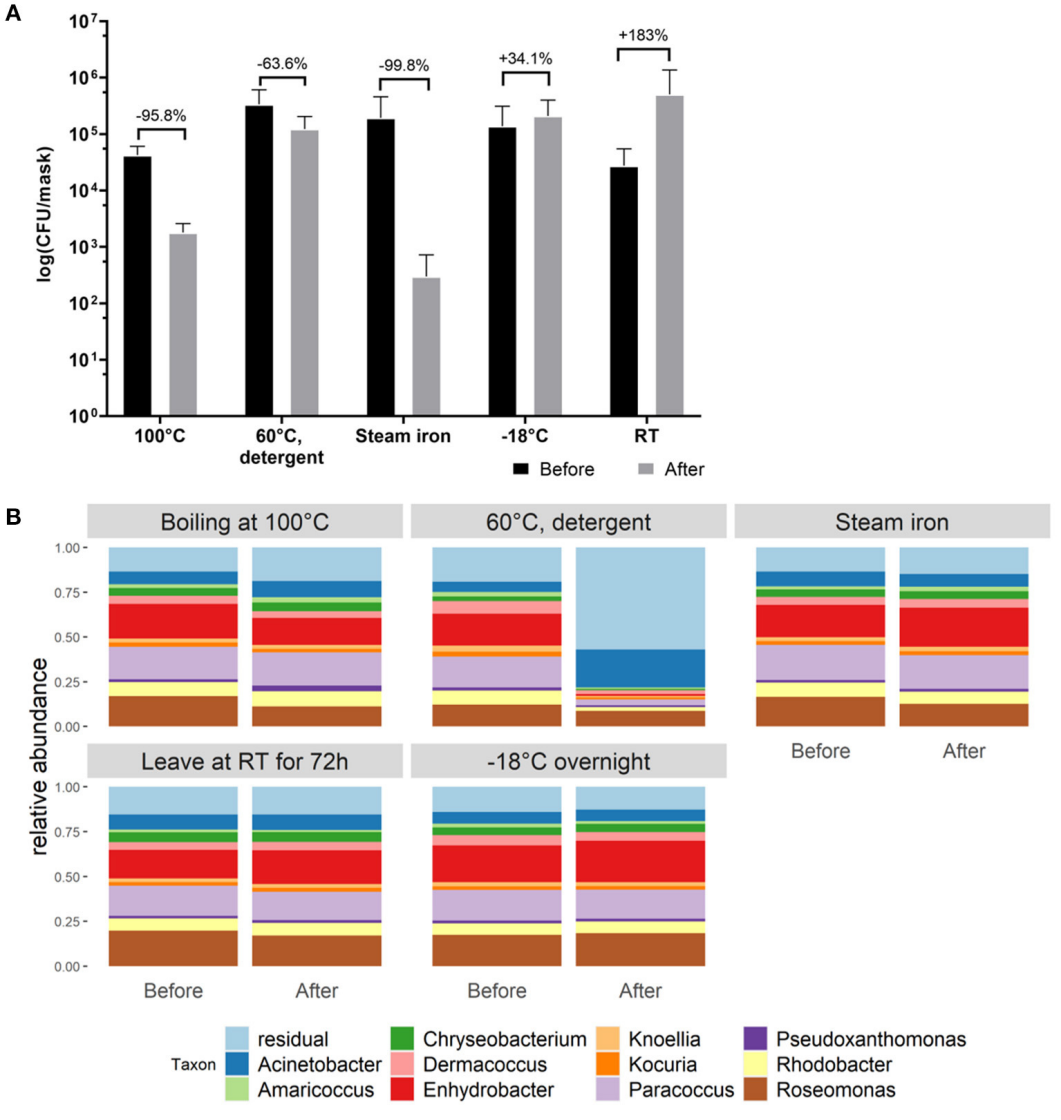
Evaluation of different cleaning methods to reduce bacterial load on cotton face masks. **(A)** Cotton mask bacterial load before and after mask cleaning. Data shown as the average CFU/mask (calculated to represent the whole face mask) after cultivation on BHI agar (cultivation on LB agar in Supplementary Figure 2). Statistics were performed using two-way ANOVA with Sidak's multiple comparisons test. No significant differences were detected. **(B)** Taxonomic bacterial community composition on the face masks at genus level for the different cleaning methods, before and after cleaning, analyzed *via* 16S rRNA gene amplicon sequencing.

In order to select the most effective method to reduce bacterial loads after wearing cotton masks, different cleaning methods were evaluated. Cleaning methods were determined as effective when a reduction in microbial biomass of 90% was established. A reduction in CFU/mask of 95.8%, 63.6% and 99.8% on both LB and BHI growth media was detected after boiling the mask at 100°C, washing at 60°C with detergent, and ironing with a steam iron, respectively. Keeping the mask overnight at −18°C and leaving the mask at room temperature for 72 h did not reduce the detected CFU counts observed after 4 h of wearing. Even more, a trend toward an increase in CFU/mask was observed by 34.1 and 183.3%, respectively, when using the latter cleaning methods (Figure 3A). The relative taxonomic abundances within the cotton face mask microbiome stayed stable after cleaning, except for washing at 60°C with detergent (Figure 3B), suggesting that this method also effectively removes the inactivated bacterial cells from the masks. *Roseomonas, Paracoccus*, and *Enhydrobacter* still dominated the face masks for more than 40% after boiling, ironing, leaving it at RT or at −18°C (Figure 3B). However, the total microbial load decreased up to over 90% for the effective cleaning methods, i.e., boiling at 100°C, washing at 60°C with detergent and ironing with a steam iron. This was expected since bacterial cells remained on the face masks after ironing and boiling as these methods kill bacterial cells, but don't remove them from the mask. Thus, DNA could still be extracted and sequenced. Washing at 60°C with detergent in a washing machine seems to be the only method that removed bacterial cells which is supported by the sequencing data (Figure 3B), where a change in relative abundances can be seen after cleaning.

### Effect of Wearing a Face Mask on the Nasal and Skin Microbiome

**Figure 4:**
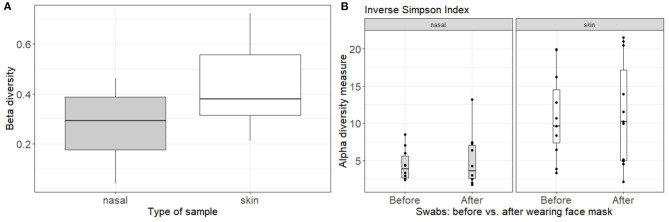
Analysis of the bacterial composition of the nasal and skin microbiome before and after wearing a face mask for 4 h. **(A)** Beta-diversity by Bray-Curtis dissimilarities, comparing the beta-diversity of the skin and nasal microbiome before and after wearing a face mask for 4 h. **(B)** Alpha-diversity by Inverse Simpson Index, grouped by time point (before and after wearing a face mask) and plots split for nasal and skin microbial samples.

In addition to studying the microbial communities of the face masks themselves, we also studied the effect of wearing the face mask for 4 h on the nasal (anterior nares) and skin (cheek) microbiome. Taxonomic microbiome profiles at ASV-level of the skin and nasal swabs are depicted in Supplementary Figure 4. Community structures for skin and nasal swabs were assessed using Bray-Curtis beta-diversity measures grouped by type of face mask (Figure 4A). The summed differences in read counts for all taxa across the different participants were analyzed with pairwise Bray-Curtis dissimilarities, comparing the beta-diversity before and after wearing the face mask. A beta-diversity of 0 indicates that the bacterial content before and after is the same, whereas a beta-diversity of 1 means that the bacterial profiles differ completely. For the skin microbial composition, a Bray-Curtis dissimilarity of 42% was observed, while this was 27% for the anterior nares, indicating that the skin microbiome profiles are somewhat more influenced by mask wearing than the nasal microbiome profiles. Additionally, alpha diversity measured by inverse Simpson is depicted in Figure 4B. No effect of wearing the face mask for 4 h on alpha-diversity in skin or nasal microbiome was observed by inverse Simpson index (*p* = 0.97 for anterior nares and *p* = 0.75 for skin).

### Survey on Mask Use and Attitudes

**Figure 5:**
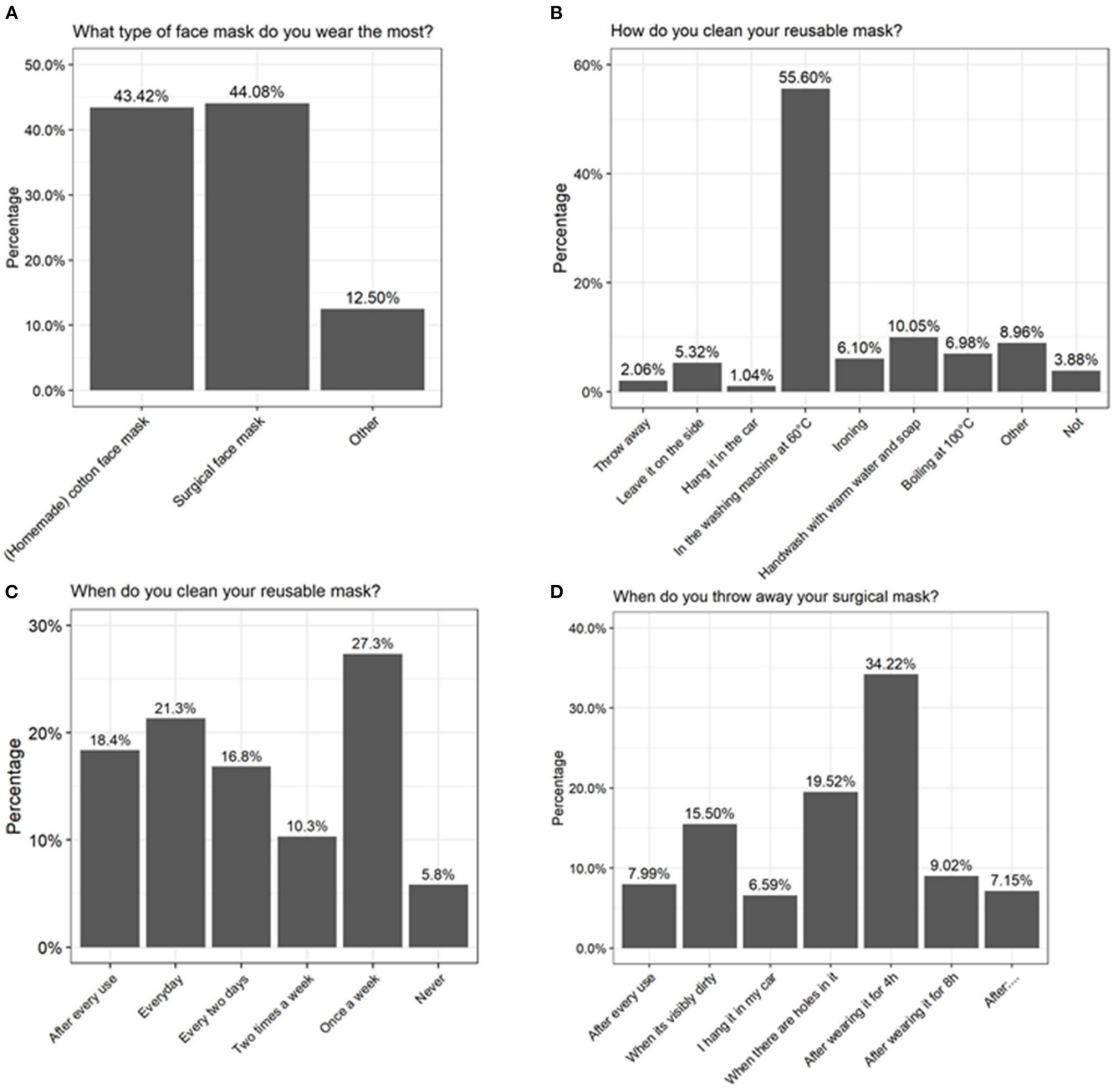
Results of a large-scale survey conducted by the University of Antwerp, filled in by over 25,000 people. Answers to the questions **(A)** “What type of face mask do you wear the most?” **(B)** “How do you clean your reusable mask?” **(C)** “When do you clean your reusable mask?” and **(D)** “When do you throw away your surgical mask?” are depicted as percentages.

To investigate whether some concerns could be raised about the mask use and hygiene in the general population, some questions were included in the 2-weekly Corona survey of the University of Antwerp on August 19, 2020. Relevant questions for this paper are listed in Supplementary Table 3. Approximately 25,000 people had filled in the questionnaire. At that moment, face masks were obligatory in public settings where a distance of 1.5 m could not be maintained, and only 21% of the responders claimed not to use a face mask daily. Regarding duration of use, 48% indicated that they did wear one for <1 h, 28% for 1-2 h, 12% for 2-4 h, 9% for 4-8 h, and 2% for more than 8 h each day. Based on the survey, no clear preference for a type of mask was observed, as 44 and 43% of the people choose to wear a surgical or cotton face masks, respectively (Figure 5A). Related to hygiene, only 8% of surgical mask users reported that they did throw it away after each use and 15% indicated that they only throw it away when it was visibly dirty or damaged (Figure 5D). Of the cotton-mask wearers, 18% indicated to wash the masks after every use independent of time, 21% performed a daily cleansing, 27% reported to only wash it once a week, and 6% indicated to never have washed their reusable face mask (Figure 5C). From all participants, also 36% claimed to have some health complaints when wearing a face mask, of which 7% reported sinusitis, 16% acne, and 77% other complaints (amongst others headache, skin irritation, and breathing difficulties).

## Discussion

While a number of studies have focused on the importance of face masks in the transmissions of respiratory viruses (2, 17, 26), accumulation of pathobionts on the masks due to human saliva and exhaled breath represents a possible underestimated biosafety concern. Microorganisms present on the skin and in the upper respiratory tract could be transferred to the face mask while wearing it. For optimal growth, bacterial cells need a surface to grow on, warmth, moisture, and nutrients, which is the environment created on the face mask due to exhaled air and water vapor (56). Growth of these microorganisms will also increase the amount of bacteria that are inhaled or could be transferred to the skin. This could theoretically cause some disturbance in the skin and nasal microbiome due to for instance the overgrowth of certain pathobionts, which are associated with an increased risk of inflammation and infections (57). For example, research has found that *S. aureus* is part of a healthy skin microbiome, but can cause skin infections when the abundance of this species increases (58). In several studies, the use of face masks has been associated with acne linked to an accumulation of *S. aureus* (59–61).

Here, we evaluated the bacterial load on cotton and surgical face masks after wearing them for 4 h, and the effects of different cleaning methods on this bacterial load and community composition. Furthermore, changes in nasal and cheek skin microbiome due to mask usage were analyzed. We detected a significant accumulation of bacteria on the cotton mask (mean of 1.48 × 10^5^ CFU/mask) as well as the surgical masks (mean of 1.98 × 10^4^ CFU/mask) after 4 h of wearing. However, these surgical masks were more sterile at the start so a larger difference in bacterial load was to be expected. Although all self-made cotton masks were cleaned beforehand, a considerable amount of bacteria was still detected (1.44 × 10^3^ CFU/mask on LB and 1.50 × 10^3^ CFU/mask on BHI). Based on our results and previous research, surgical masks appear to be the better option regarding bacterial load accumulation when masks need to be worn for at least 4 h (62). This could be due to the lower water retention of surgical masks compared to cotton masks, as well as their better ventilation properties (56). The latter results in a lower temperature inside the mask, which, together with lower humidity levels, is a less ideal environment for bacterial growth and so a lower bacterial growth compared to cotton masks (56).

Cotton or cloth masks are known to be a good substrate for microbial growth and to hold moisture very well, which is in line with the results obtained in this study and is also observed by others (32). Zhiqing et al. have shown that the bacterial count on face masks of surgeons was directly proportional with the operating time (62). Therefore, we hypothesize that this bacterial load will be even greater after 8 h, which is the maximal wearing time recommended by the government. However, people indicated to wear it even more than the recommended 8 h.

In addition to the bacterial load, also the bacterial composition is an important factor to consider. Here, we evaluated both the microbiome on the mask, as well as changes on the nasal and cheek microbiome before and after wearing a cotton mask. The latter has—to the best of our knowledge—never been examined before. In this study, the microbiome on cotton face masks after 4 h of wearing was mainly represented by *Roseomonas, Paracoccus*, and *Enhydrobacter* taxa, whereas the surgical face mask microbiome consisted mostly of *Streptococcus* and *Staphylococcus* taxa, which were also found on the cotton masks, albeit in much lower abundances. Our cultivation results detected specific strains belonging to genera such as *Staphylococcus* spp., *Bacillus* spp., *Acinetobacter* spp. that are known to be associated with the skin and respiratory tract (49, 63). For example for *Staphylococcus* spp., *S. epidermidis, S. warneri*, and *S. caprae* are known as commensals on the human skin, maintaining the healthy skin (46). However, *S. epidermidis* and *S. aureus* are also known as pathobionts causing inflammatory skin conditions such as atopic dermatitis and acne vulgaris (64–68). *S. aureus* is also a colonizer of the airways due to the expression of surface adhesins and is known as a commensal bacterium as well as an important pathogen, causing respiratory tract infections (69, 70). Additionally, *Acinetobacter* spp. is also considered a part of the healthy human skin and respiratory microbiome (48, 49). However, other members of this genus, such as *Acinetobacter baumanni*, can cause wound infections and pneumonia (49).

We also tested the accumulation of antibiotic-resistant strains on the face masks, as antibiotic-resistant strains are a worldwide problem and it is believed that by 2050 more people will die from an antibiotic-resistant bacterial infection than from cancer (52). Especially *S. aureus* and *A. baumanni* are part of the *Enterococcus faecium, Staphylococcus aureus, Klebsiella pneumoniae, Acinetobacter baumannii, Pseudomonas aeruginosa*, and *Enterobacter* species (ESKAPE) pathogens, which is a group of bacteria that causes life-threatening nosocomial infections and are characterized by potential drug resistance mechanisms (55). Approximately 43% of selected colonies were resistant to at least one of the two tested antibiotics (ampicillin and erythromycin) and 6% was even resistant to both. Erythromycin is a macrolide antibiotic active against many respiratory pathogens (50). However, due to the extensive use of macrolide antibiotics, respiratory pathogens show increasing resistance to macrolides (51, 54). The same is true for ampicillin, a beta-lactam antibiotic equivalent to amoxicillin in terms of activity (71, 72). In our study, 21.3 and 38.5% of the isolates was resistant to ampicillin and erythromycin, respectively. In the future, larger microbiological studies including different age and socioeconomic groups are warranted to extrapolate our findings to a larger population.

Considering the high detected bacterial load, it is important that surgical masks are disposed and that cotton masks are disinfected properly after each use. Our analysis indicated that boiling at 100°C, washing at 60°C with detergent, or ironing with a steam iron are most effective in reducing microbial load on cotton face masks. In general, we observed a considerable survival of *Bacillus spp*. after cleaning the cotton face masks. *Bacillus spp*. are spore-forming bacteria and are therefore more resistant to environmental stress factors such as heat (73). In addition, washing at 60°C with detergent was the only method that removed bacterial DNA on the face masks, which was reflected in our sequencing data since the microbial profile changed (Figure 2B). Boiling at 100°C and ironing did significantly inactivate the present bacteria (Figure 3A), but amplicon sequencing showed that the bacterial DNA remained on the face masks after cleaning (Figure 2B). These methods are in line with the Belgian government regulations to wash a cotton face mask every 8 h or after 4 h of intensive use. However, in the survey done by the University of Antwerp, Belgium, it was clear that not everyone followed these recommendations. Only the minority of survey participants (39%) claimed to wash their cotton face mask after every use or every day (Figure 5B).

In addition to culture-based approaches, we also analyzed the impact of wearing a face mask (cotton and surgical) on the nasal and skin microbiome by DNA-based analysis of swabs. Since there was only 4 h in between the sampling points (before and after wearing the face mask), we hypothesized no significant effect on the microbiome would be observed. Indeed, wearing a cotton or surgical mask for 4 h did not significantly influence specific taxa of the nasal or skin microbiome. Also for the Bray-Curtis beta diversity analysis, no difference was observed, although a trend toward a change in community structures of the skin microbiome could be observed. In theory, this change could imply a negative or beneficial effect on the microbiome. However, based on other clinical research regarding the effect of face masks on the skin, we expect that the change in microbiome composition would rather be unfavorable (59–61, 74). More large-scale longitudinal microbiome analyses are thus needed to investigate whether there might be an association of a wearing a mask and changes in the skin and/or nasal microbiome when worn for longer time periods.

## Conclusions

Bacteria, and specifically pathobionts, accumulate on both surgical and more so on cotton face masks after 4 h of wearing. When the same face masks are worn for longer periods of time, surgical masks might be a better option due to a lower bacterial load. In addition, surgical face masks should probably best be disposed of after every use and cotton face masks should be properly sterilized. The latter can be efficiently done by boiling at 100°C, washing at 60°C with detergent, and ironing using a steam iron. More research is required to investigate whether mask use beyond 4 h could lead to a dysbiosis in the skin and nasal microbiome and be associated to conditions such as acne. This research emphasizes that face masks should be better evaluated to weigh the risks of disease transmission rate against other biosafety risks such as bacterial overgrowth, especially in vulnerable populations and in situations where physical distancing and proper ventilation are available.

## Data Availability Statement

The datasets presented in this study can be found in online repositories. The names of the repository/repositories and accession number(s) can be found below: https://www.ebi.ac.uk/ena/browser/view/PRJEB45406?show=reads.

## Ethics Statement

The studies involving human participants were reviewed and approved by Ethical committee University Hospital Antwerp (UZA). The patients/participants provided their written informed consent to participate in this study.

## Author Contributions

Data-analyses and visualization was performed by EC, LD, and WV. Data interpretation was done by EC, LD, IS, ID, DV, IC, and SL. Ethical committee submission was done by EC, LD, ID, VV, and SL. Special thanks to the Research Foundation Flanders and KP for making it possible to include questions relevant for this research in the Great Corona survey. The original draft was written by EC, LD, and SL. All authors worked on the conceptualization of the research projects, contributed to reviewing and editing of the paper, and proofread and approved the manuscript.

## Conflict of Interest

The authors declare that the research was conducted in the absence of any commercial or financial relationships that could be construed as a potential conflict of interest.

## Publisher's Note

All claims expressed in this article are solely those of the authors and do not necessarily represent those of their affiliated organizations, or those of the publisher, the editors and the reviewers. Any product that may be evaluated in this article, or claim that may be made by its manufacturer, is not guaranteed or endorsed by the publisher.
