# Supplementary material for: Cotton and Surgical Face Masks in Community Settings: Bacterial Contamination and Face Mask Hygiene
Source: Front Med (Lausanne). 2021 Sep 3;8:732047. doi: 10.3389/fmed.2021.732047 (PMC8446422; doi:10.3389/fmed.2021.732047)
Supplement: Supplementary file 1 [file Data_Sheet_1.docx]

Supplementary Material

# Supplementary Data

# Supplementary Figures and Tables

## Supplementary Figures

##
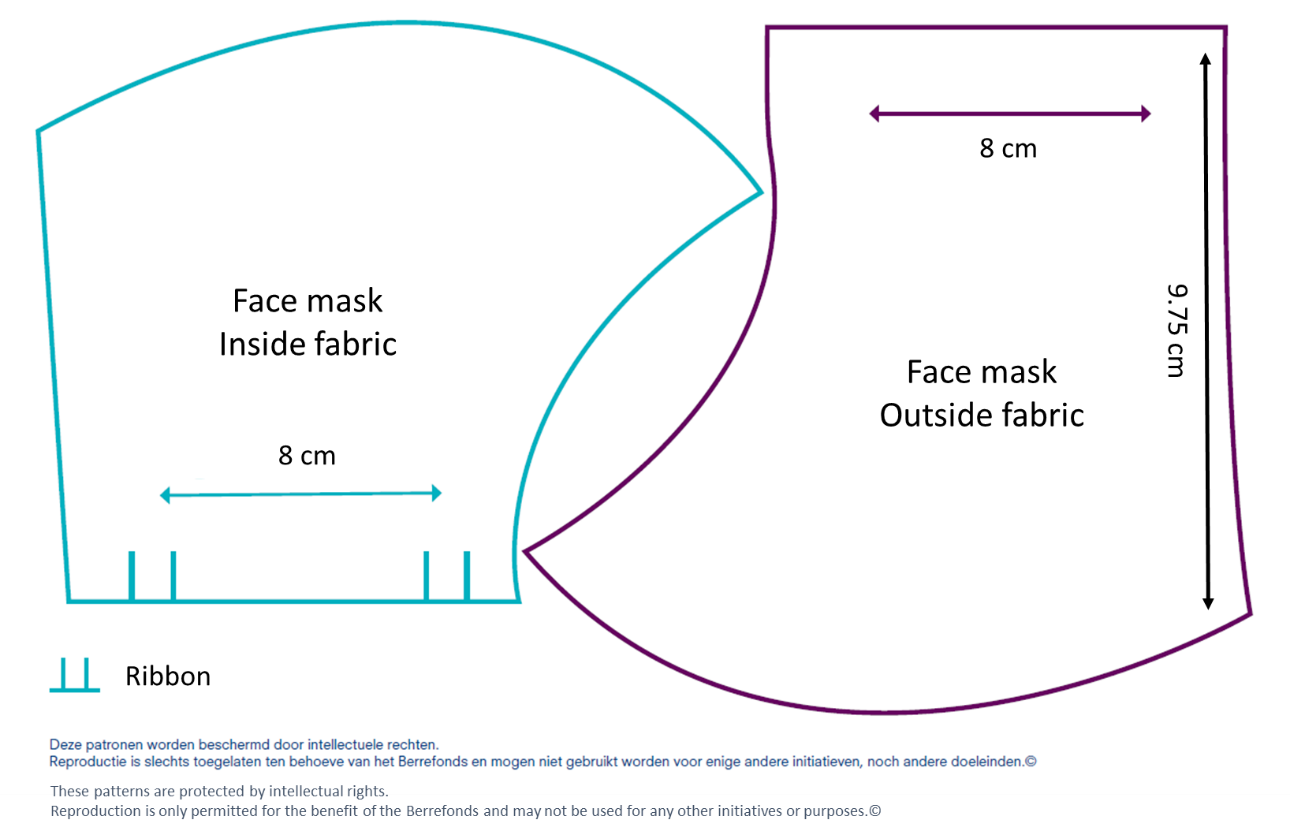


## Supplementary Figure 1. Stitching pattern for self-made cotton masks used in this study.


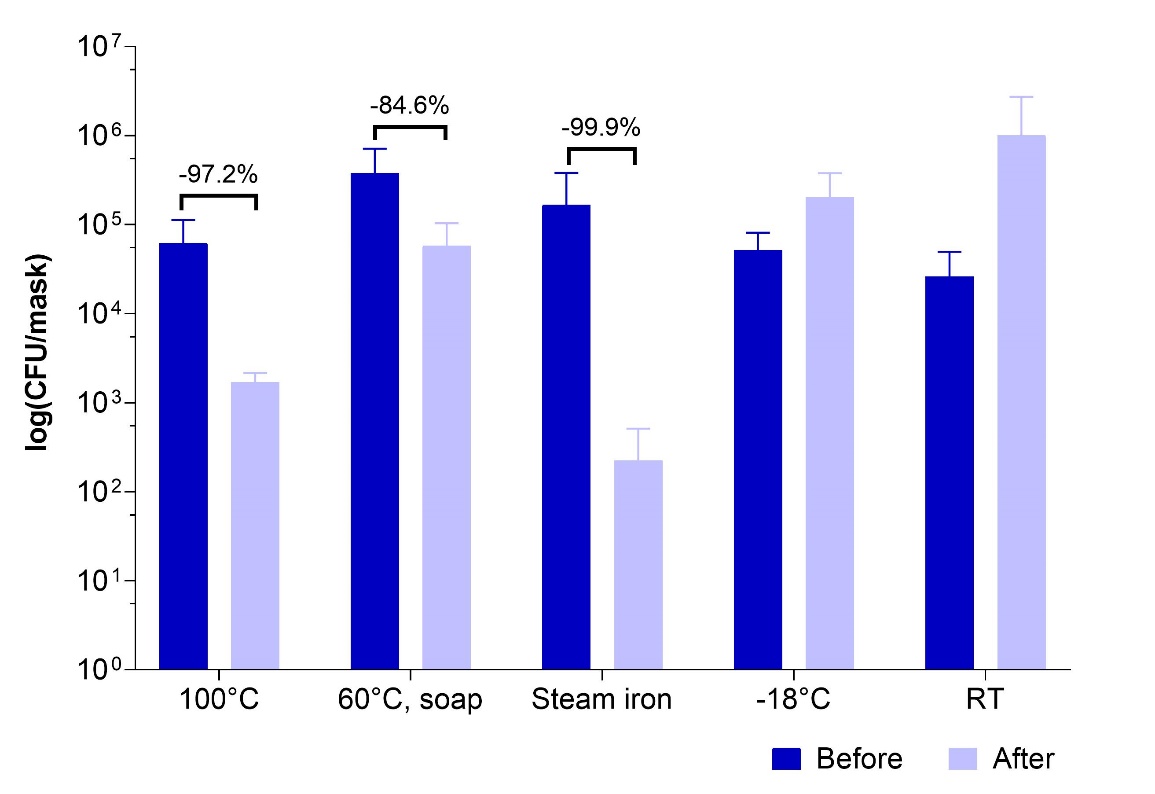


**Supplementary Figure 2**. **Evaluation of different cleaning methods to reduce bacterial load on cotton face masks.** Evaluation of microbial load on cotton face masks before and after cleaning via culturing on LB and BHI agar plates. The graph shows the average CFU/mask (calculated to represent the whole face mask) after cultivation on LB-agar. Different cleaning methods: 100°C = soaking in boiling water for a few minutes, 60°C = washing machine at 60°C with detergent, Steam iron = ironing with a steam iron for appr. 2 minutes, Freezer = leaving overnight in the freezer (avg. -18°C), 72h = leaving at room temperature for 72h.


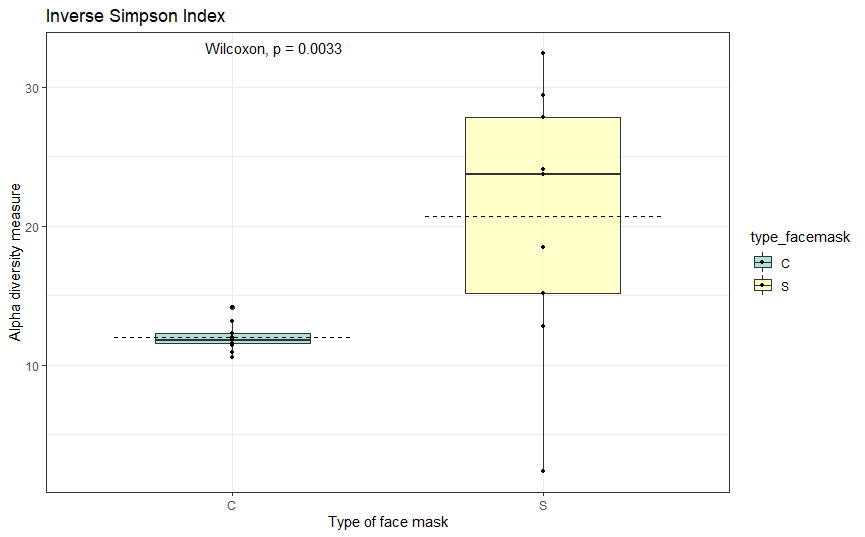


**Supplementary Figure 3**. **Difference in alpha-diversity on cotton (C) and surgical (S) face masks.** Alpha-diversity is indicated by Inverse Simpson Index (richness and evenness). C = cotton face mask, S = surgical face mask.


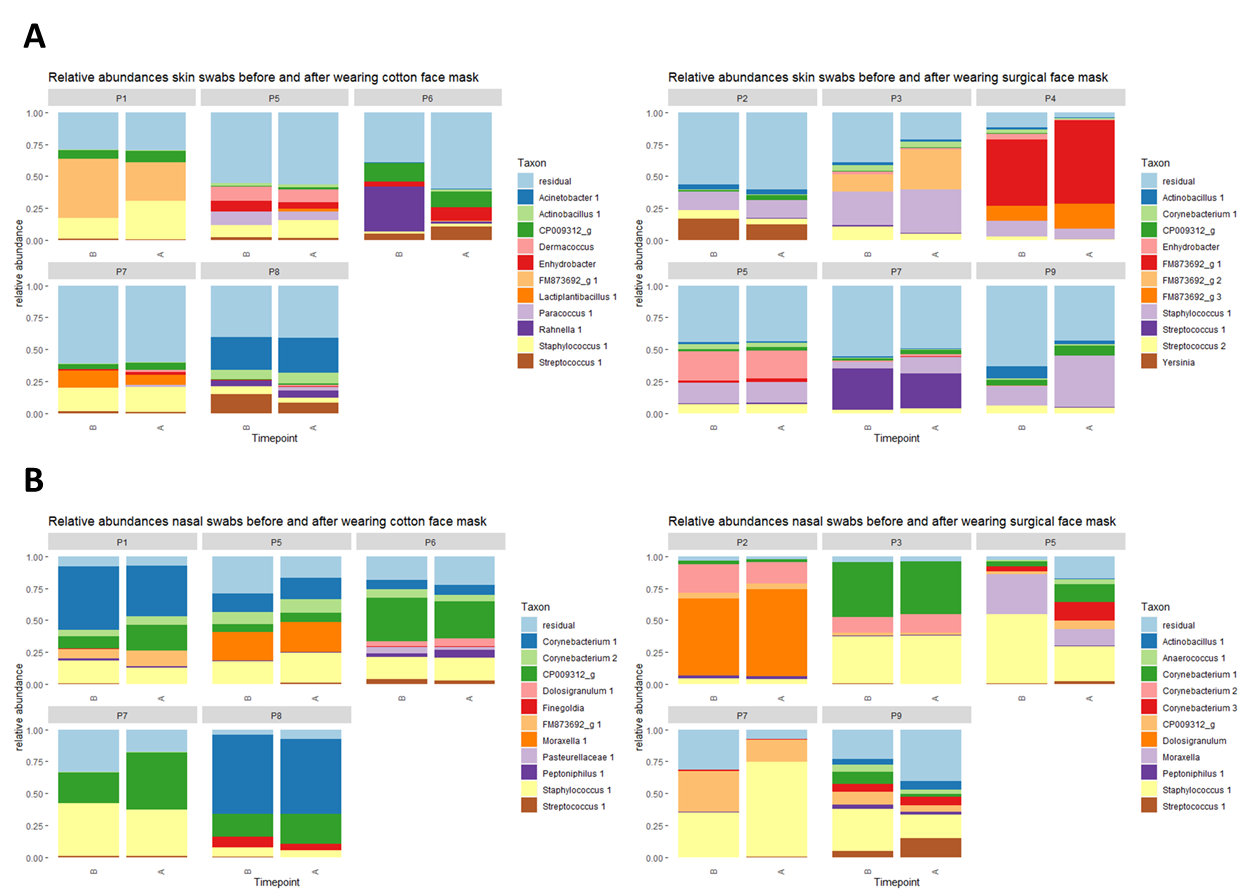


**Supplementary Figure 4**. **Analysis of the bacterial composition of the nasal and skin microbiome before and after wearing a face mask for 4h.** (A) Taxonomic bacterial community composition on the skin (cheek) swabs at ASV-level for each individual, analyzed via 16S rRNA amplicon sequencing. Left: Participants who wore cotton face mask. Right: Participants who wore a surgical face mask. (B) Taxonomic bacterial community composition on the nasal swabs at ASV-level for each individual, analyzed via 16S rRNA amplicon sequencing. Left: Participants who wore cotton face mask. Right: Participants who wore a surgical face mask.

## Supplementary Tables

**Supplementary Table 1. Sanger sequencing and antibiotic resistance results**

| **Colony** | **Strain** | **Growth medium** | **Resistance to ampicillin** | **Resistance to erythromycin** |
| --- | --- | --- | --- | --- |
| **1** | *Bacillus thuringiensis* | LB | + |  |
| **2** | *Acinetobacter* sp. | LB |  |  |
| **3** | *Staphylococcus* sp. | LB |  |  |
| **4** | *B. cereus* | LB | + |  |
| **5** | *Acinetobacter* sp. | LB |  |  |
| **6** | *B. thuringiensis* | LB | + |  |
| **7** | *B. thuringiensis* | LB | + |  |
| **8** | *B. toyonensis* | LB | + |  |
| **9** | *Staphylococcus epidermidis* | LB |  | + |
| **10** | *A. septicus* | LB |  | + |
| **11** | *Pantoea agglomerans* | LB | + |  |
| **12** | *S. caprae* | LB |  |  |
| **13** | *S. epidermidis* | LB |  | + |
| **14** | *S. epidermidis* | LB |  |  |
| **15** | *Bacillus* sp. | LB |  |  |
| **16** | *S. epidermidis* | LB |  |  |
| **17** | *S. epidermidis* | LB | + |  |
| **18** | *S. aureus* | LB |  |  |
| **19** | *B. paramycoides* | LB | + | + |
| **20** | *B. cereus* | LB | + | + |
| **21** | *S. epidermidis* | LB |  |  |
| **22** | *B. thuringiensis/cereus* | LB | + |  |
| **23** | *B. wiedmannii* | LB | + | + |
| **24** | *S. epidermidis* | LB |  |  |
| **25** | *B. cereus* | BHI |  |  |
| **26** | *S. epidermidis* | BHI |  |  |
| **27** | */* | BHI |  |  |
| **28** | *Acinetobacter* sp. | BHI |  |  |
| **29** | *Bacillus* sp. | BHI | + |  |
| **30** | *B. thuringiensis* | BHI | + |  |
| **31** | *S. epidermidis* | BHI |  | + |
| **32** | *A. septicus* | BHI |  |  |
| **33** | *S. warneri* | BHI |  |  |
| **34** | *S. epidermidis* | BHI |  | + |
| **35** | *B. cereus* | BHI |  |  |
| **36** | *S. epidermidis* | BHI |  |  |
| **37** | *S. epidermidis* | BHI |  | + |
| **38** | *B. cereus* | BHI |  |  |
| **39** | *S. epidermidis* | BHI |  |  |
| **40** | *S. warneri* | BHI |  |  |
| **41** | *B. safensis* | BHI |  |  |
| **42** | *S. aureus* | BHI |  |  |
| **43** | *Bacillus* sp. | BHI | + |  |
| **44** | *Bacillus* sp. | LB |  |  |
| **45** | *Lysinibacillus* sp. | BHI |  |  |
| **46** | *B. cereus* | BHI |  |  |

**Supplementary Table 2.** **Composition of the used culturing media**

| **Lysogeny broth (LB) medium^1^** | **Brain heart infusion (BHI) medium^2^** |
| --- | --- |
| 10.0 g/L tryptone | 17.5 g/L brain-heart infusion solids (Porcine) |
| 5.0 g/L yeast extract | 10.0 g/L tryptose |
| 5.0 g/L sodium chloride | 2.0 g/L glucose |
|  | 5.0 g/L sodium chloride |
|  | 2.5 g/L disodium hydrogen phosphate |

^1^Sigma-Aldrich, Saint Louis, Missouri, USA ; ^2^LAB M, Lancashire, UK

**Supplementary Table 3.** **Questions from the large-scale survey relevant for this study and their possible answers**

| **Question number** | **Question** | **Possible answers** |
| --- | --- | --- |
| 1 | What is your year of birth? |  |
| 2 | Which gender do you identify with? | - Male - Female - Other |
| 3 | Where do you live (postal code)? |  |
| 4 | Are you staying in Belgium at the moment? | - Yes - No |
| 5 | What is your highest educational degree (if you are a student, give the degree you are studying for)? | - Primary education - Secondary education - “hoge school” - University - Post-university - Other |
| 6 | Do you live alone or do you have house mates? | - I live alone - I have house mates |
| 7 | Which face mask do you wear most often? | - Surgical facemask - (Homemade) cotton facemask - (Homemade) knitted facemask - (Homemade) synthetic face mask - (Homemade) facemask (material unknown) - Scarf - Bandana - FFP2 mask - Other |
| 8 | When the answer to question 7 was ‘surgical facemask’: When do you throw away your face mask? | - After every use - When it’s visibly dirty - After 4 h of wearing - After 8 h of wearing - When its broken/there are holes in it/... - “I hang it in my car” - “I throw it away after:” |
| 9 | When the answer to question 7 was **not** ‘surgical facemask’: How often do you wash your reusable fae mask | - After every use - Every day - Every two days - Twice a week - Once a week - Never |
| 10 | How do you disinfect your facemask? | - In the washing machine at 60°C - Boiling - Ironing - Throw away - Put away - Hang it up in the car - Hand washing with water and soap - Other: … - Not |
| 11 | When you are not using your facemask, where do you keep it? | - In a plastic bag - In an envelope - In a box - In your pocket - In your bag - On your chin |
| 12 | How many hours do you wear your facemask on a regular day? | - Not, less than 1 h - 1-2 h - 2-4 h - 4-8 h - More than 8 h |
| 13 | Do you suffer from any complaints when wearing a facemask? | - Yes - No |
| 14 | If the answer to question 13 was ‘yes’: You are having complaints. Can you assign which ones? | - Sinusitus since wearing facemasks - Acne since wearing facemasks - Other: ... |
| 15 | Some questions about displacements   - Do you still move by foot in places where a facemask is obliged? - Do you still move by bike in places where a facemask is obliged? - How much do you use your car? - How much do you use public transportation? | - Never - Less than before (only for essential things) - The same as before - More than before - Not applicable |
| 16 | Do you go less to places where facemasks are obliged?   - Supermarkets/grocery stores - Other stores - Cinema - Amusement parks - Zoos - Public spaces (libraries, town hall,…) - Parks/forrests | - Yes - No - Not applicable |
| 17 | In which ways do you feel that a facemask hinders your daily life? Multiple answers are possible | - Not - I am hearing impaired/deaf so I have a hard time understanding people - I miss non-verbal communication - Facemasks give me a claustrophobic feeling - I do not go outside as much, which influences my wellbeing - I do less sport which influences my well being - Other: |
